# Trends and Disparities in Mortality Involving Pancreatic Cancer and Pulmonary Embolism in the United States, 1999–2024: A CDC WONDER Analysis

**DOI:** 10.3390/diseases14080303

**Published:** 2026-08-20

**Authors:** Arkadeep Dhali, Jyotirmoy Biswas, Ali Shan Hafeez, Sajjad Ahmed Khan, Saikat Mandal

**Affiliations:** 1 Academic Unit of Gastroenterology, Sheffield Teaching Hospitals NHS Foundation Trust, Sheffield S10 2JF, UK; 2 School of Medicine, Dentistry and Biomedical Sciences, Queen’s University Belfast, Belfast BT9 7BL, UK; 3 Department of Medicine, Barasat Government Medical College and Hospital, Barasat 700124, India; biswasjyotirmoy2001@gmail.com; 4 Department of Medicine, CMH Multan Institute of Medical Sciences, Multan 60000, Pakistan; alishanhaffeez@gmail.com; 5 Department of Internal Medicine, Birat Medical College Teaching Hospital, Morang 56613, Nepal; 6 School of Medicine, University of Nottingham, Nottingham NG7 2UH, UK

**Keywords:** pancreatic neoplasms, pulmonary embolism, venous thromboembolism, mortality, cause of death, United States

## Abstract

Background: U.S. mortality trends involving pancreatic cancer and pulmonary embolism (PE) and whether PE is becoming increasingly represented among pancreatic cancer deaths remain incompletely characterized. Methods: We analyzed CDC WONDER Multiple Cause of Death data for adults aged ≥ 25 years from 1999 through 2024. Deaths were included in the primary analysis when pancreatic cancer and PE were both recorded as underlying or contributing causes. Age-adjusted mortality rates (AAMRs) were evaluated using Joinpoint Regression Program version 6.0.1. Forecasting and additional weighted log-linear and segmented regression analyses were performed in R version 4.5. Additional analyses evaluated race/ethnicity, the proportion of pancreatic cancer underlying-cause deaths with PE recorded, and sensitivity to the COVID-19 years. Results: Overall, 18,243 deaths involved both pancreatic cancer and PE. The AAMR increased from 0.19 per 100,000 in 1999 to 0.55 in 2024. Mortality increased by 2.65% annually during 1999–2016 and by 8.54% annually during 2016–2024, with a significantly steeper post-2016 slope (*p* < 0.001). Rates were consistently higher among males and non-Hispanic Black individuals. Hispanic or Latino mortality increased significantly during 2011–2024, whereas estimates for Asian or Pacific Islander individuals were too sparse or frequently suppressed to support reliable longitudinal trend analysis. Among deaths with pancreatic cancer as the underlying cause, the proportion with PE recorded increased from 1.00% in 1999 to 2.85% in 2024, with significantly faster growth after 2016 (*p* < 0.001). Excluding 2020–2021 did not materially alter the post-2016 increase. Conclusions: Mortality involving pancreatic cancer and PE increased substantially in the United States, and PE was recorded in a growing proportion of pancreatic cancer deaths. These death-certificate data identify an increasing population-level burden but cannot establish causality or demonstrate the effectiveness of specific clinical interventions.

## 1. Introduction

Pancreatic cancer is a highly lethal gastrointestinal malignancy whose mortality burden continues to rise in the United States. Population-based studies have demonstrated persistent differences in pancreatic cancer incidence and mortality by sex, race/ethnicity, geography, and urbanization [1,2]. More recent U.S. mortality analyses extending into the 2020s indicate that this burden has not plateaued [3]. These disparities are clinically important because pancreatic ductal adenocarcinoma (PDAC) is also strongly associated with venous thromboembolism (VTE), a complication that may further worsen outcomes.

In a prospective multicenter cohort of 731 patients with newly diagnosed PDAC, Frere et al. reported that 20.8% developed VTE during follow-up; VTE was associated with shorter progression-free and overall survival [4]. Pulmonary embolism (PE), a potentially fatal manifestation of VTE, may be present even at initial cancer staging. In a retrospective cohort of 174 treatment-naïve patients with biopsy-proven PDAC, incidental PE was detected in 5.7% at staging chest computed tomography, with significantly greater occurrence among patients with T4 tumors [5]. These observations are consistent with both the prothrombotic biology and advanced disease burden of PDAC.

Pancreatic cancer is particularly relevant to investigation alongside PE because it is consistently recognized as one of the most thrombogenic malignancies [6]. Unlike many other solid tumors, pancreatic cancer induces a profound hypercoagulable state through tumor-derived tissue factor expression, platelet activation, inflammatory cytokines, endothelial dysfunction, and release of procoagulant extracellular vesicles [7]. These mechanisms substantially increase VTE risk. At the same time, modern pancreatic cancer care has changed through more intensive multidrug chemotherapy, broader use of cross-sectional imaging, and evolving thromboprophylaxis strategies, each of which could affect the population of patients at risk or the ascertainment of PE [8,9,10,11,12].

Previous studies have characterized pancreatic cancer mortality or thromboembolic complications separately, and a recent U.S. analysis identified an acceleration in PE mortality among patients with cancer after 2016 [13]. However, U.S. mortality trends involving both pancreatic cancer and PE remain insufficiently defined. Importantly, it remains unclear whether increasing mortality involving both conditions merely parallels the underlying pancreatic cancer burden or whether PE is being recorded in an increasing proportion of pancreatic cancer deaths. We therefore examined U.S. mortality records from 1999 to 2024 to evaluate temporal, demographic, and geographic patterns, expand race/ethnicity analyses where data permitted, quantify PE as a proportion of pancreatic cancer deaths, assess the robustness of the post-2016 trend to the COVID-19 period, and forecast the future mortality trajectory.

## 2. Methods

### 2.1. Study Design and Data Source

This retrospective, population-based study used publicly available mortality data from the Centers for Disease Control and Prevention Wide-ranging Online Data for Epidemiologic Research (CDC WONDER) Multiple Cause of Death files. These files contain an underlying cause of death and contributing causes recorded on U.S. death certificates. Because the data are publicly available, deidentified, and aggregate, institutional review board approval and informed consent were not required. The study followed STROBE reporting guidance [14].

### 2.2. Case Definition

Deaths were included in the primary analysis when both pancreatic cancer and pulmonary embolism were recorded anywhere on the death certificate as either underlying or contributing causes. Pancreatic cancer was identified using ICD-10 codes C25.0, C25.1, C25.2, C25.3, C25.4, C25.7, C25.8, and C25.9. PE was identified using ICD-10 codes I26.0 and I26.9. This definition identifies mortality involving both conditions and does not establish that PE caused death or determine the temporal relationship between the two diagnoses.

The primary analytic period was 1999 through 2024. Urbanization analyses were limited to 1999 through 2020 because the CDC WONDER mortality source used for 1999–2020 provided the applicable urban–rural classification variable, whereas a comparable urbanization variable was unavailable for the 2021–2024 records used in this analysis. State-level estimates were presented separately for 1999–2020 and 2021–2024 to correspond with the relevant CDC WONDER source-file periods.

### 2.3. Study Variables and Outcomes

The study population included U.S. decedents aged 25 years and older. Mortality was analyzed by year, sex, race/ethnicity, U.S. Census region, state, and urbanization status. Stable full-period race-specific Joinpoint analyses were performed for non-Hispanic Black or African American and non-Hispanic White decedents. Hispanic or Latino decedents were additionally evaluated over 2011–2024, the first contiguous interval with reportable annual AAMRs suitable for trend analysis. Asian or Pacific Islander and American Indian or Alaska Native estimates were also examined; however, annual estimates were frequently suppressed or based on sparse counts, precluding reliable longitudinal trend analysis. Therefore, APCs were not estimated for these groups. No suppressed values were imputed.

The primary outcome was the annual age-adjusted mortality rate per 100,000 population for deaths involving both pancreatic cancer and PE. Rates were standardized to the 2000 U.S. standard population using the direct method [15]. Secondary outcomes included subgroup-specific mortality patterns, state-level variation, and forecasted mortality rates. To determine whether the increase in mortality involving both conditions simply reflected increasing pancreatic cancer mortality or increasing PE involvement, an additional proportional analysis was performed. For this analysis, pancreatic cancer was required to be the underlying cause of death, and the numerator comprised those deaths in which PE was also recorded among the multiple causes. The annual proportion was calculated as the number of pancreatic cancer underlying-cause deaths with PE recorded divided by the total number of pancreatic cancer underlying-cause deaths.

### 2.4. Statistical Analysis

Temporal trends in AAMRs were analyzed using Joinpoint Regression Program version 6.0.1. Annual rates and their standard errors were modeled on the log scale. Joinpoint regression estimated annual percent change (APC) and average annual percent change (AAPC) with 95% confidence intervals. The number and location of joinpoints were selected using permutation-based model selection; therefore, the reported time-period divisions were data-driven rather than defined a priori. A two-sided *p* value < 0.05 was considered statistically significant. To evaluate whether the overall post-2016 slope differed significantly from the pre-2016 slope, an additional weighted segmented log-linear model with a knot at 2016 was fitted to the annual overall AAMRs.

For the additional Hispanic or Latino series, exploratory weighted log-linear regression of log AAMR on calendar year was used to estimate the APC because the shorter series was not considered suitable for full Joinpoint modeling. The analysis accounted for the precision of annual AAMRs using inverse-variance weighting. APCs were not estimated for Asian or Pacific Islander or American Indian or Alaska Native populations because the available annual estimates were too sparse or frequently suppressed for reliable trend estimation.

Forecasting was performed using autoregressive integrated moving average (ARIMA) models. Annual AAMRs for each analytic stratum were treated as separate univariate time series. The auto.arima() function in the R forecast package (Version 9.0.2) was used to identify the preferred ARIMA specification by minimizing the corrected Akaike information criterion (AICc) [16,17]. First-order differencing and drift terms were permitted when supported by the selected model. Model adequacy was assessed by inspection of residual behavior and Ljung–Box tests for residual autocorrelation.

Additional sensitivity and secondary analyses were performed in R version 4.5. First, the post-2016 overall trend was re-estimated after excluding 2020 and 2021 to assess whether the COVID-19 period accounted for the observed acceleration. Second, the annual proportion of pancreatic cancer underlying-cause deaths with PE recorded was analyzed using segmented binomial regression, with 2016, the breakpoint identified in the primary overall Joinpoint analysis, used as the knot. Annual changes before and after 2016 and the difference between the two slopes were evaluated. Ten-year ARIMA forecasts were generated for the overall, sex-specific, race-specific, and Census-region series; urbanization forecasts were generated from the available 1999–2020 series.

## 3. Results

### 3.1. Overall Mortality Trends

**Figure 1:**
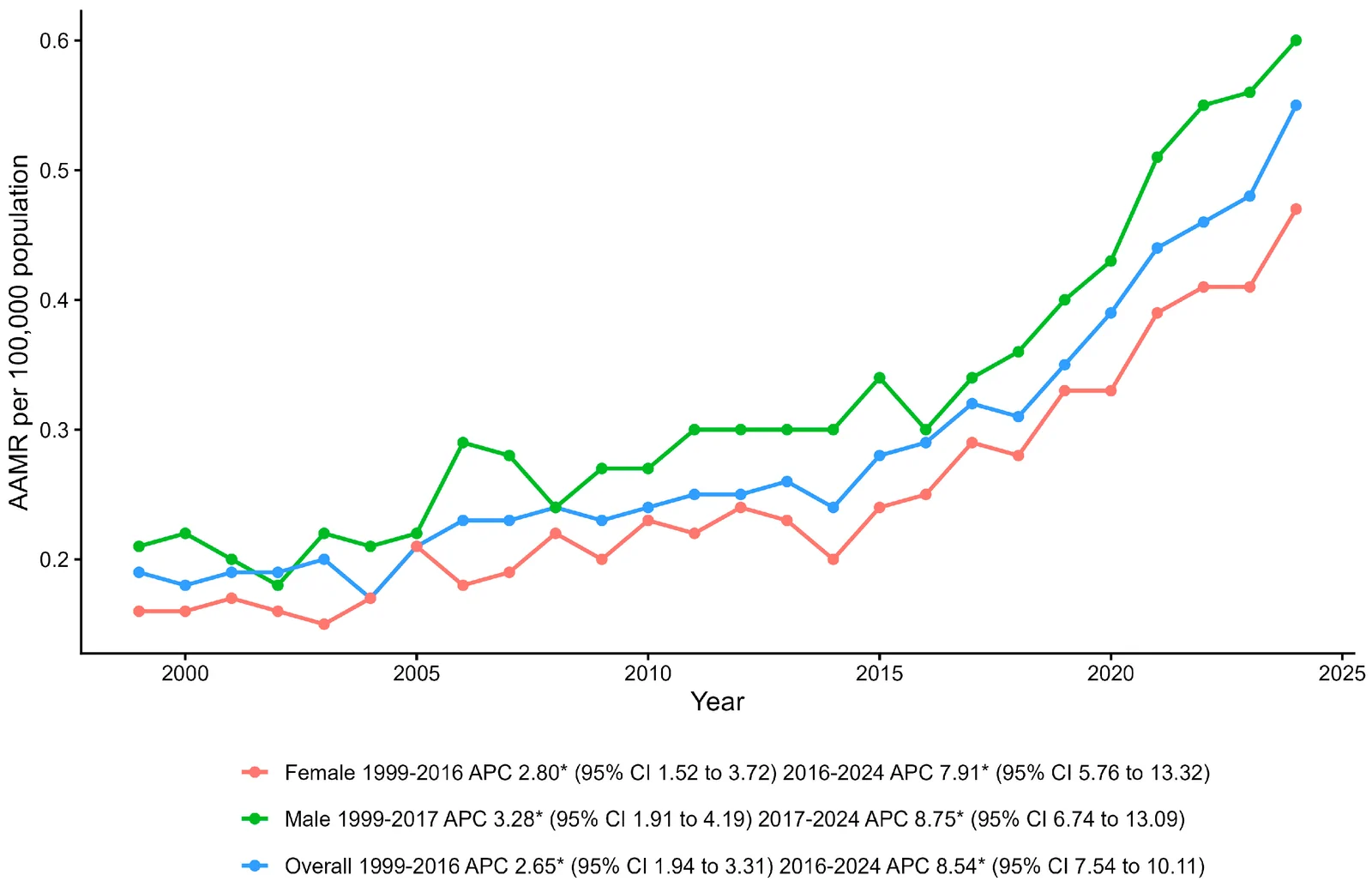
Overall and sex-stratified age-adjusted mortality rates for deaths involving pancreatic cancer and pulmonary embolism per 100,000 population in the United States, 1999–2024. * Indicates that the annual percentage change (APC) is significantly different from zero at α = 0.05. AAMR, age-adjusted mortality rate; APC, annual percent change; CI, confidence interval.

From 1999 to 2024, there were 18,243 deaths in the United States involving both pancreatic cancer and pulmonary embolism among adults aged 25 years and older. The overall age-adjusted mortality rate increased from 0.19 per 100,000 in 1999 to 0.55 per 100,000 in 2024 (Figure 1).

Joinpoint regression identified one statistically supported inflection point in 2016. Mortality increased significantly from 1999 to 2016 (APC 2.65% per year; 95% CI 1.94 to 3.31) and subsequently increased more rapidly from 2016 to 2024 (APC 8.54%; 95% CI 7.54 to 10.11). Across the full study period, the AAPC was 4.50% (95% CI 4.22 to 4.87). In an additional weighted segmented analysis, the post-2016 slope was significantly steeper than the pre-2016 slope (*p* < 0.001). After excluding 2020–2021, the post-2016 increase remained 8.43% per year (95% CI 7.33 to 9.53; *p* < 0.001), indicating that the acceleration was not explained solely by the peak COVID-19 years.

### 3.2. Sex-Specific Trends

Mortality rates were consistently higher among males than females throughout the study period. From 1999 to 2024, the AAMR increased from 0.21 to 0.60 per 100,000 among males and from 0.16 to 0.47 per 100,000 among females (Figure 1).

Among females, AAMRs increased significantly from 1999 to 2016 (APC 2.80%; 95% CI 1.52 to 3.72), followed by an accelerated increase from 2016 to 2024 (APC 7.91%; 95% CI 5.76 to 13.32). Among males, AAMRs increased from 1999 to 2017 (APC 3.28%; 95% CI 1.91 to 4.19), followed by a steeper increase from 2017 to 2024 (APC 8.75%; 95% CI 6.74 to 13.09). Full-period AAPCs were significantly increasing for both females (4.41%; 95% CI 3.83 to 5.06) and males (4.78%; 95% CI 4.26 to 5.34).

### 3.3. Race/Ethnicity-Specific Trends

**Figure 2:**
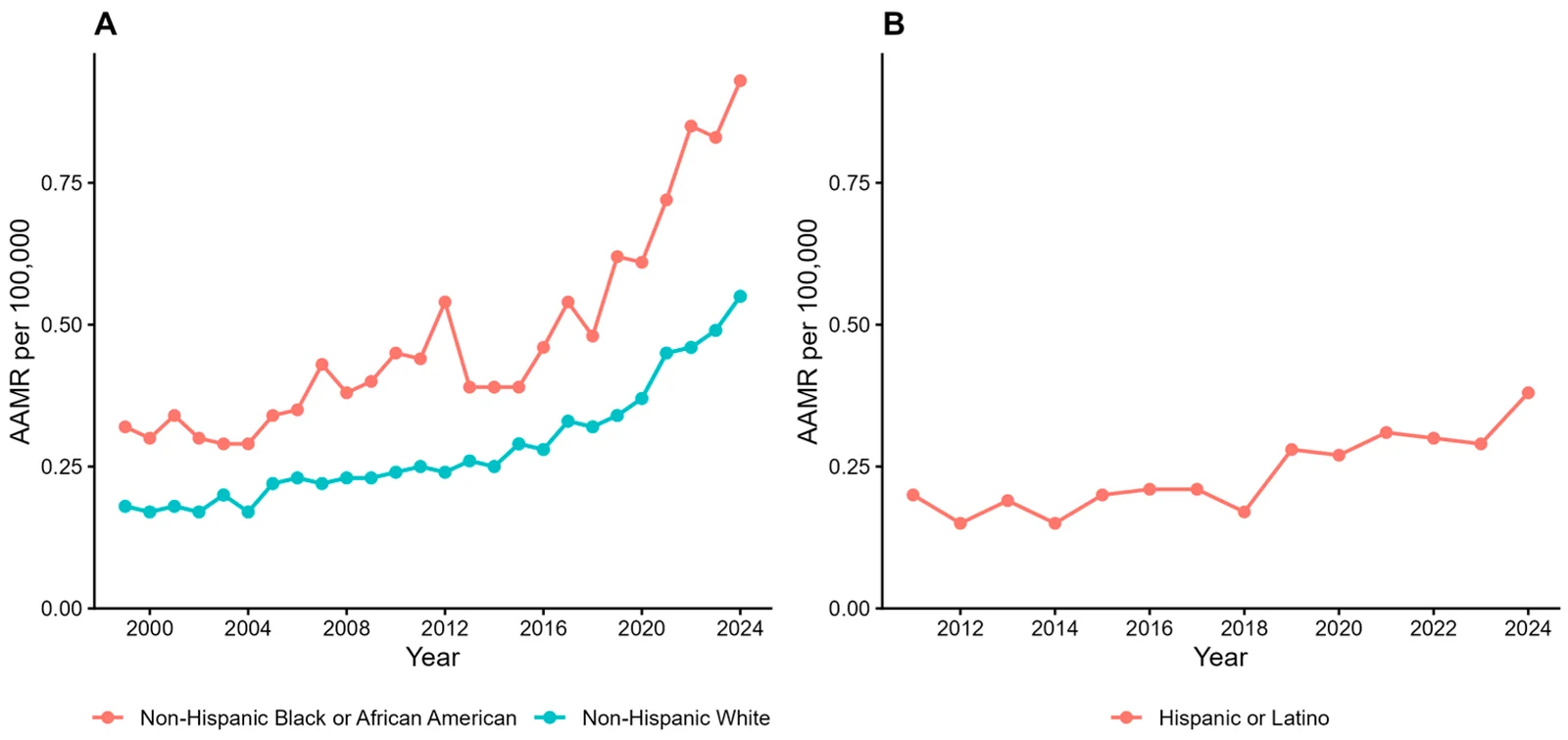
Age-adjusted mortality rates for deaths involving pancreatic cancer and pulmonary embolism by race/ethnicity. (**A**) shows non-Hispanic Black or African American and non-Hispanic White individuals, 1999–2024. (**B**) shows Hispanic or Latino individuals during 2011–2024, corresponding to the contiguous interval with reportable annual estimates suitable for trend analysis. Asian or Pacific Islander and American Indian or Alaska Native estimates were too sparse or frequently suppressed for reliable longitudinal trend modeling.

Race/ethnicity-stratified analyses demonstrated persistent disparities (Figure 2). Among groups with stable full-period estimates, non-Hispanic Black or African American individuals had the highest AAMRs, increasing from 0.32 per 100,000 in 1999 to 0.93 per 100,000 in 2024. Among non-Hispanic White individuals, AAMRs increased from 0.18 to 0.55 per 100,000 over the same period. In the additional analysis, the Hispanic or Latino AAMR increased from 0.20 per 100,000 in 2011 to 0.38 in 2024, with an estimated annual increase of approximately 6.3% (95% CI approximately 4.5 to 8.1; *p* < 0.001).

Among non-Hispanic Black or African American individuals, mortality increased significantly from 1999 to 2012 (APC 4.11%; 95% CI 2.08 to 21.17), showed no statistically significant change from 2012 to 2015 (APC −5.33%; 95% CI −10.38 to 13.97), and subsequently increased sharply from 2015 to 2024 (APC 10.11%; 95% CI 3.91 to 17.11). Among non-Hispanic White individuals, AAMRs increased significantly from 1999 to 2018 (APC 3.33%; 95% CI 2.77 to 3.89) and more rapidly from 2018 to 2024 (APC 9.81%; 95% CI 7.65 to 13.57). Full-period mortality increased significantly in both groups. Asian or Pacific Islander estimates were also examined; however, annual estimates were frequently suppressed or based on sparse counts, precluding reliable longitudinal trend analysis. Therefore, no APC or Joinpoint trend was estimated for this group. American Indian or Alaska Native estimates were similarly insufficient for stable longitudinal trend modeling. The available estimates are reported in Supplementary Table S1.

### 3.4. Regional Trends

**Figure 3:**
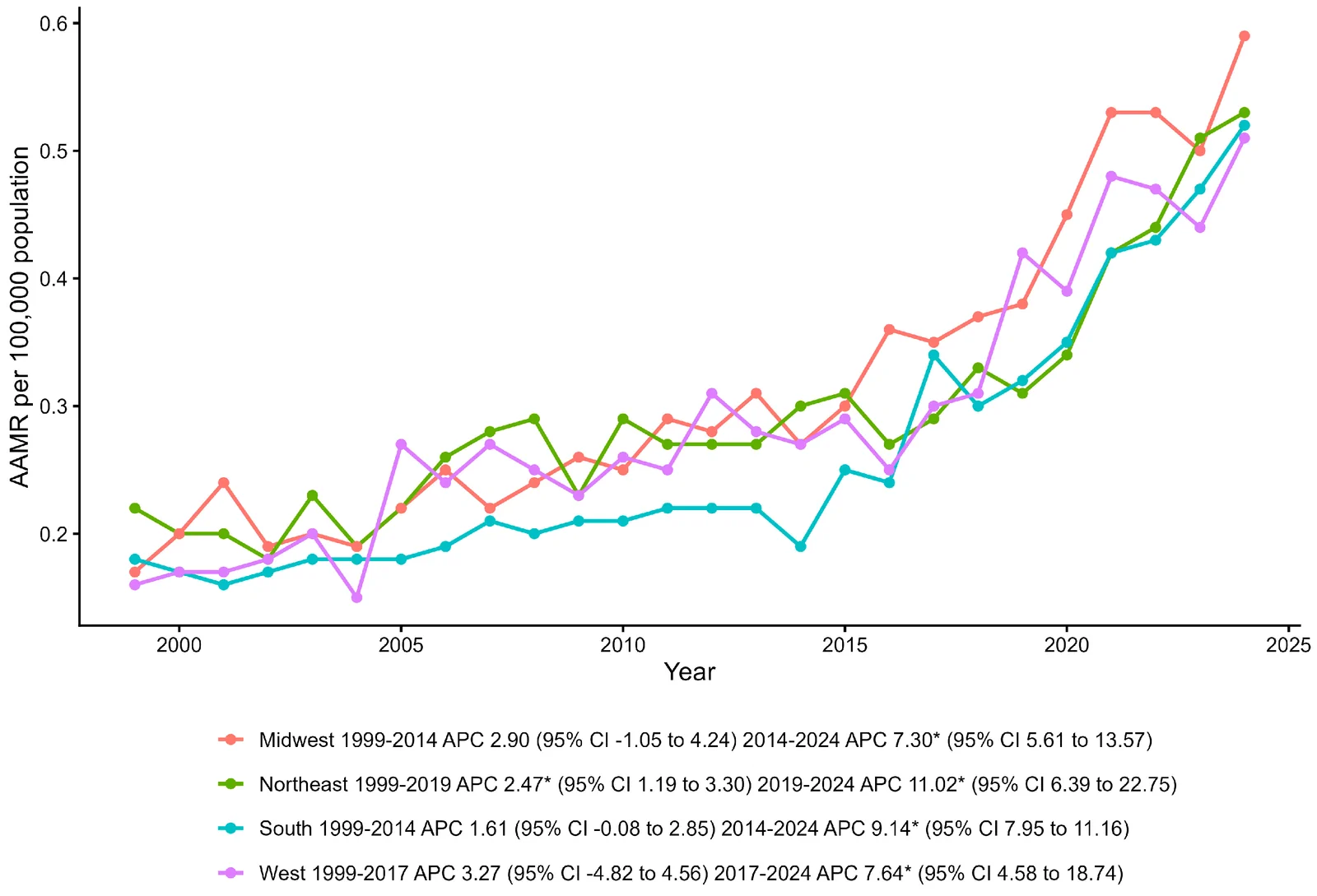
Age-adjusted mortality rates for deaths involving pancreatic cancer and pulmonary embolism per 100,000 population, stratified by U.S. Census region, United States, 1999–2024. * Indicates that the annual percentage change (APC) is significantly different from zero at α = 0.05. AAMR, age-adjusted mortality rate; APC, annual percent change; CI, confidence interval.

Mortality increased significantly across all U.S. Census regions over the full study period (Figure 3). In 2024, the highest AAMR was observed in the Midwest at 0.59 per 100,000, followed by the Northeast at 0.53, South at 0.52, and West at 0.51. From 1999 to 2024, AAMRs increased from 0.17 to 0.59 per 100,000 in the Midwest, from 0.22 to 0.53 in the Northeast, from 0.18 to 0.52 in the South, and from 0.16 to 0.51 in the West.

Full-period mortality increased significantly in every Census region, with the largest AAPC in the Midwest (4.64%; 95% CI 3.95 to 5.40), followed by the South (4.56%; 95% CI 4.02 to 5.19), West (4.48%; 95% CI 3.34 to 5.45), and Northeast (4.13%; 95% CI 3.48 to 4.74). The data-driven joinpoints occurred at different times by region, 2014 in the Midwest and South, 2017 in the West, and 2019 in the Northeast, indicating heterogeneous timing of acceleration rather than a single synchronized national breakpoint.

### 3.5. Urbanization Trends

**Figure 4:**
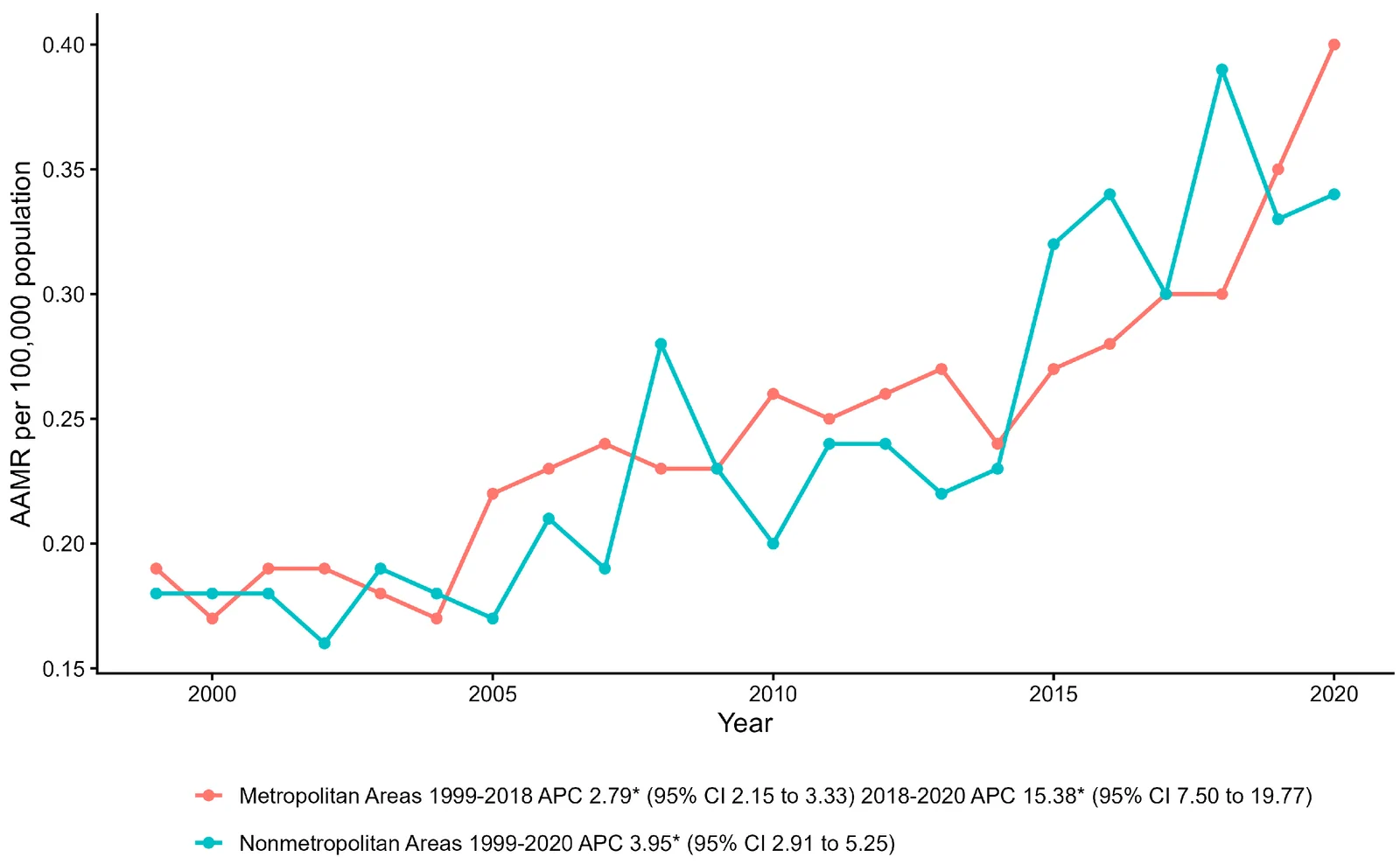
Age-adjusted mortality rates for deaths involving pancreatic cancer and pulmonary embolism per 100,000 population, stratified by urbanization, United States, 1999–2020. * Indicates that the annual percentage change (APC) is significantly different from zero at α = 0.05. AAMR, age-adjusted mortality rate; APC, annual percent change; CI, confidence interval.

Urbanization analyses were limited to 1999 through 2020 because comparable urban–rural classification data were unavailable for the later source records. During this interval, AAMRs increased from 0.19 to 0.40 per 100,000 in metropolitan areas and from 0.18 to 0.34 per 100,000 in nonmetropolitan areas (Figure 4).

In metropolitan areas, AAMRs increased significantly from 1999 to 2018 (APC 2.79%; 95% CI 2.15 to 3.33), followed by a marked acceleration from 2018 to 2020 (APC 15.38%; 95% CI 7.50 to 19.77). In nonmetropolitan areas, AAMRs increased significantly throughout 1999–2020 without an identified joinpoint (APC 3.95%; 95% CI 2.91 to 5.25). The short 2018–2020 metropolitan segment should be interpreted cautiously because the detected joinpoint represents a statistical change in slope rather than evidence of a discrete causal event.

### 3.6. State-Level Variation

**Figure 5:**
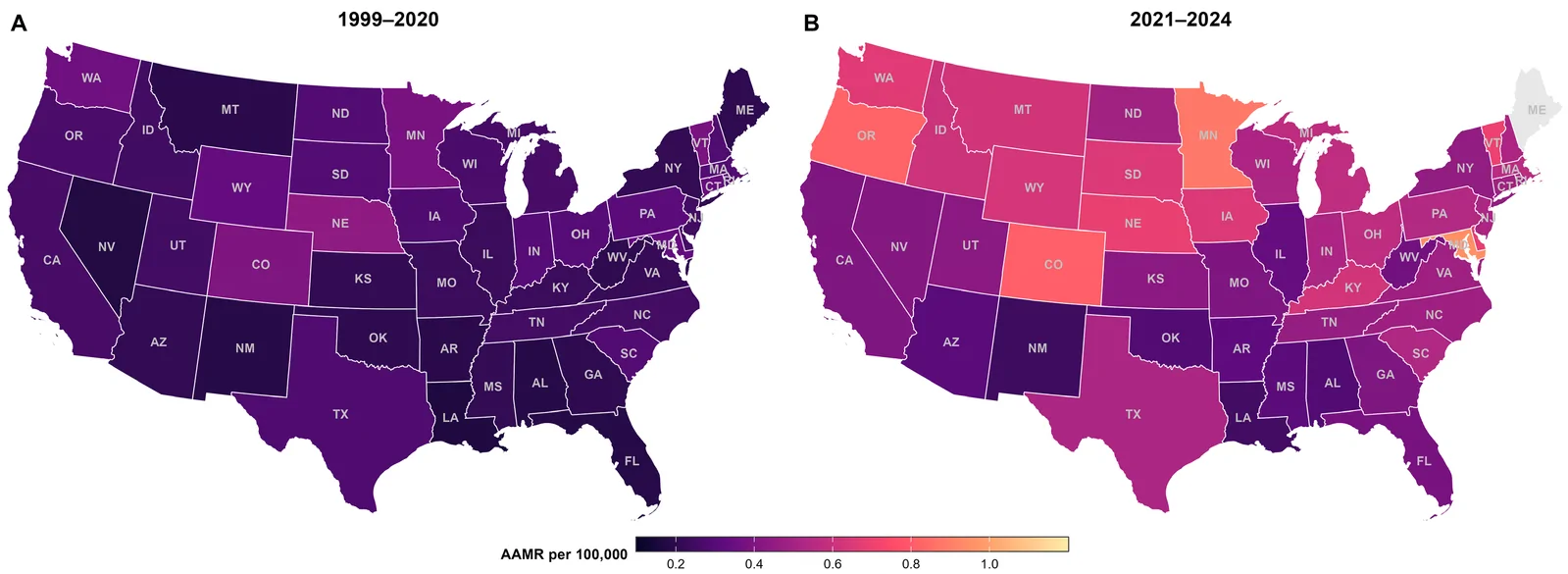
Age-adjusted mortality rates for deaths involving pancreatic cancer and pulmonary embolism per 100,000 population, stratified by state, United States, 1999–2020 and 2021–2024. State postal abbreviations are displayed on the maps; corresponding full state names are provided in Supplementary Table S3. Maine is shown in gray because its 2021–2024 estimate was suppressed. (**A**) Age-adjusted mortality rate stratified by state from 1999-2020. (**B**) Age-adjusted mortality rate stratified by state from 2021-2024.

State-level mortality varied substantially across both analytic periods (Figure 5). During 1999–2020, the highest AAMRs were observed in Nebraska (0.44 per 100,000), the District of Columbia (0.42), Colorado (0.40), and Minnesota and Vermont (0.38), whereas the lowest rates were observed in Louisiana (0.16) and Nevada (0.17). During 2021–2024, the highest AAMRs were observed in the District of Columbia (1.14 per 100,000), Maryland (0.95), Minnesota (0.88), Oregon (0.82), and Colorado (0.81). Maine’s recent-period estimate was suppressed.

### 3.7. Forecasted Mortality Trends

**Figure 6:**
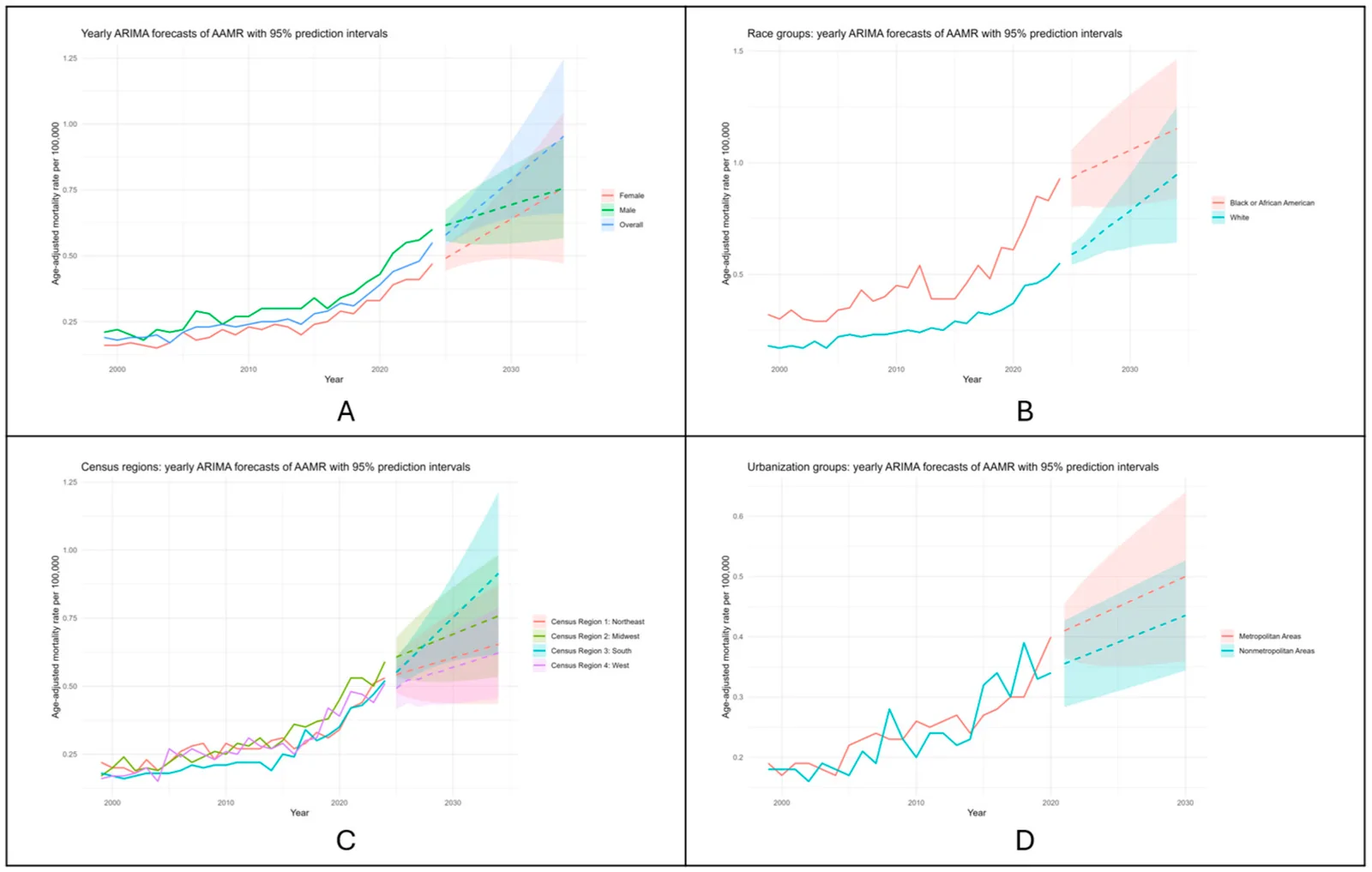
Observed and ARIMA-forecasted age-adjusted mortality rates for deaths involving pancreatic cancer and pulmonary embolism in the United States, overall and stratified by sex, race, U.S. Census region, and urbanization. Shaded areas represent 95% prediction intervals. Forecasts represent extrapolations of observed temporal patterns rather than causal predictions. (**A**) Age-adjusted mortality rate stratified by sex. (**B**) Age-adjusted mortality rate stratified by race. (**C**) Age-adjusted mortality rate stratified by U.S. census region. (**D**) Age-adjusted mortality rate stratified by urbanization.

ARIMA-based projections indicated that mortality involving pancreatic cancer and PE may continue to increase over the forecast period. The overall AAMR was projected to reach 0.95 per 100,000 by 2034 (Figure 6).

Among females, the projected age-adjusted mortality rate was 0.76 per 100,000 by 2034, while the corresponding projected rate among males was also 0.76 per 100,000. Race-specific forecasts indicated a projected 2034 age-adjusted mortality rate of 1.15 per 100,000 among non-Hispanic Black or African American individuals, compared with 0.95 per 100,000 among non-Hispanic White individuals.

Among Census regions, the highest projected age-adjusted mortality rate in 2034 was observed in the South at 0.91 per 100,000, followed by the Midwest at 0.76, Northeast at 0.65, and West at 0.62. Urbanization forecasts through 2030 projected age-adjusted mortality rates of 0.50 per 100,000 in metropolitan areas and 0.44 per 100,000 in nonmetropolitan areas. Residual diagnostic testing did not identify significant residual autocorrelation in the selected ARIMA models; all Ljung–Box tests were nonsignificant (*p* > 0.05). Detailed diagnostic results are provided in the Supplementary Material.

### 3.8. Pulmonary Embolism as a Proportion of Pancreatic Cancer Deaths

**Figure 7:**
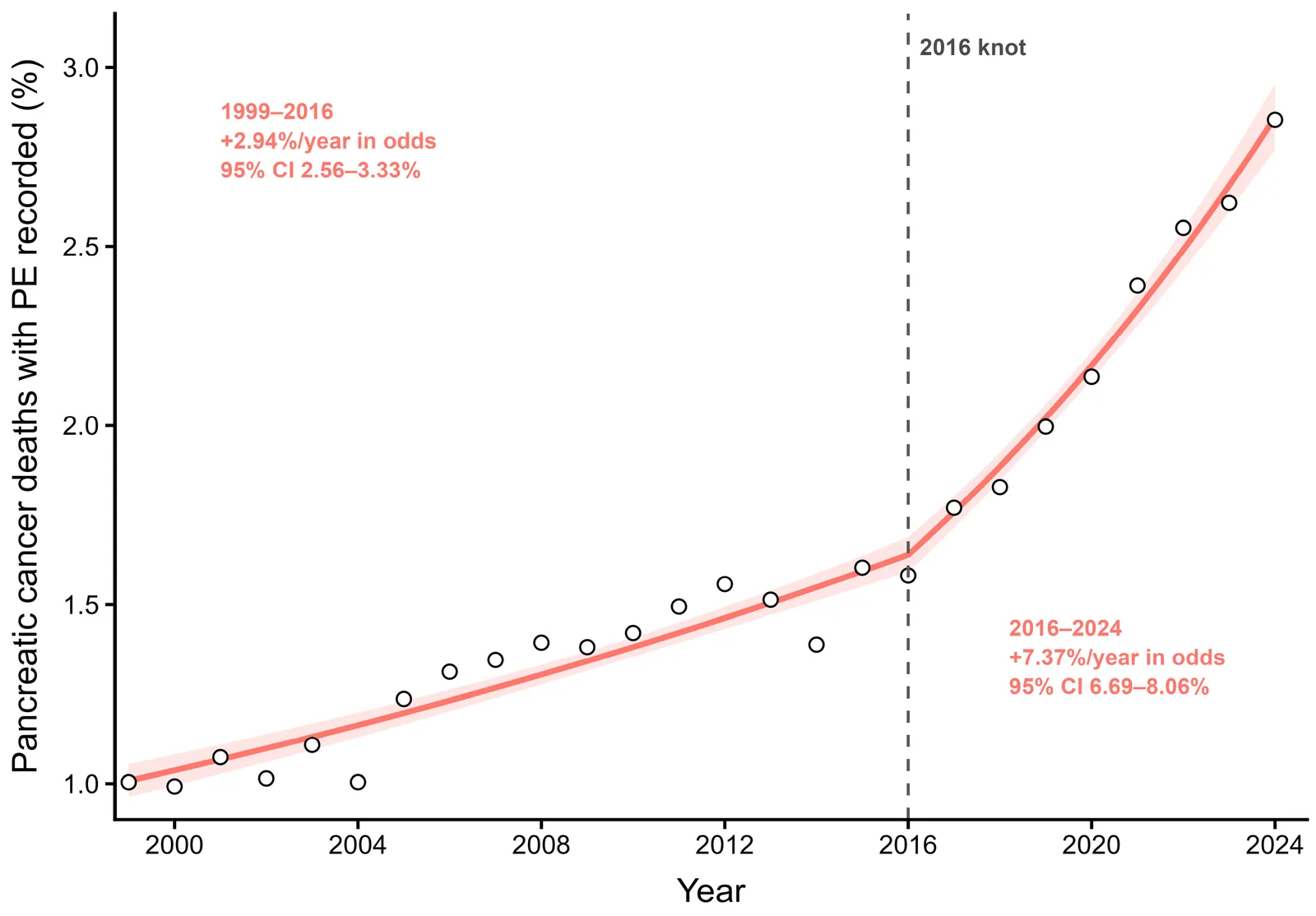
Proportion of pancreatic cancer underlying-cause deaths with pulmonary embolism recorded, United States, 1999–2024. Circles represent the observed annual proportions of deaths with pancreatic cancer as the underlying cause in which pulmonary embolism (PE) was recorded among the multiple causes. The solid line represents fitted values from segmented binomial regression with 2016, the breakpoint identified in the primary overall AAMR analysis, used as the knot; the shaded band represents the corresponding 95% confidence interval. The vertical dashed line indicates the 2016 knot. The annual odds of PE being recorded increased by 2.94% per year before 2016 (95% CI, 2.56–3.33%) and by 7.37% per year after 2016 (95% CI, 6.69–8.06%), with a significantly steeper post-2016 slope (*p* < 0.001).

In the secondary analysis restricted to deaths with pancreatic cancer as the underlying cause, the pancreatic cancer AAMR increased comparatively modestly from 16.41 per 100,000 in 1999 to 17.53 per 100,000 in 2024. Over the same period, the proportion of pancreatic cancer deaths in which PE was recorded anywhere on the death certificate increased from 1.00% in 1999 (292 of 29,077 pancreatic cancer deaths) to 2.85% in 2024 (1464 of 51,313). In segmented binomial regression using 2016 as the knot identified in the primary overall Joinpoint analysis, the annual odds of PE being recorded increased by 2.94% per year before 2016 (95% CI 2.56 to 3.33) and by 7.37% per year after 2016 (95% CI 6.69 to 8.06). The increase was significantly steeper after 2016 (*p* < 0.001). Thus, mortality involving PE increased not only as a population rate but also as a proportion of deaths for which pancreatic cancer was the underlying cause (Figure 7).

## 4. Discussion

In this U.S. population-based analysis, the AAMR for mortality involving pancreatic cancer and PE increased from 0.19 per 100,000 in 1999 to 0.55 in 2024, with a statistically supported acceleration after 2016. Mortality remained higher among males, and non-Hispanic Black individuals had the highest rates among racial groups with stable full-period estimates. The additional proportional analysis materially strengthens interpretation of the temporal trend: among deaths for which pancreatic cancer was the underlying cause, PE was recorded in 1.00% in 1999 and 2.85% in 2024, while the pancreatic cancer AAMR itself changed much more modestly. Therefore, the increasing joint mortality rate cannot be explained solely by a parallel increase in pancreatic cancer mortality. Nevertheless, death-certificate data cannot determine whether the increasing proportion reflects greater PE incidence, improved detection, changes in treatment or survival, altered case mix, changes in death certification, or a combination of these factors.

The marked post-2016 acceleration should not be interpreted as evidence of a single causal event. The latter part of the study period coincided with substantial evolution in pancreatic cancer treatment and supportive care. FOLFIRINOX and gemcitabine plus nab-paclitaxel established more active multidrug systemic regimens, and more recent combinations have further expanded treatment options [10,11,12]. Improvements in cancer survival, even when modest, may increase the duration during which patients remain exposed to active malignancy, chemotherapy, central venous access, hospitalization, and other thrombotic risks. Conversely, increased awareness of cancer-associated thrombosis and evolving thromboprophylaxis strategies may reduce some VTE events. These competing influences make a single mechanistic explanation for the observed population-level trend unlikely.

Anticoagulation practice provides another important context. Pancreatic cancer is classified as a very-high-risk tumor site in the Khorana model [18], and randomized trials in pancreatic cancer have demonstrated reductions in thromboembolic events with low-molecular-weight heparin prophylaxis [19,20]. More broadly, randomized studies of apixaban and rivaroxaban have supported thromboprophylaxis in selected high-risk ambulatory patients with cancer [8,9]. However, prophylaxis is necessarily individualized because bleeding risk, thrombocytopenia, invasive procedures, comorbidity, and patient preferences limit universal treatment. The increasing mortality signal observed here therefore should not be interpreted as evidence that existing prophylaxis is ineffective or that broader anticoagulant use would necessarily reduce population-level mortality.

The COVID-19 pandemic warrants specific consideration because SARS-CoV-2 infection is associated with thrombotic complications and because the pandemic disrupted cancer diagnosis and treatment pathways [21,22]. In the present series, however, the acceleration began in 2016, several years before the pandemic. Moreover, exclusion of 2020–2021 yielded a post-2016 annual increase of 8.43% (95% CI 7.33 to 9.53), closely resembling the primary estimate. COVID-19 may have influenced individual years through thromboinflammation, delayed presentation, altered health-care utilization, or changes in death certification, but the sensitivity analysis indicates that the pandemic years alone do not explain the onset or persistence of the late-period increase.

Changes in imaging and PE ascertainment are also biologically and clinically plausible contributors. Contemporary pancreatic cancer care relies heavily on cross-sectional staging and interval imaging, and incidental PE can be detected even in treatment-naïve patients [5]. Increasing diagnostic intensity may therefore increase recognition of clinically silent emboli and the likelihood that PE is documented on the death certificate. Because ICD-10 coding was used throughout the study period, the post-2016 acceleration cannot be attributed to a simple ICD-9-to-ICD-10 transition; however, changes in diagnostic ascertainment, documentation, and death-certification practices may still have contributed.

The increasing mortality observed in this study should be considered in the context of broader trends in both pancreatic cancer and cancer-associated PE. Previous US population-based studies have demonstrated increasing pancreatic cancer mortality and persistent differences by sex, race and geography [1,2]. A recent analysis of PE mortality across all cancers similarly identified an inflection in 2016- the annual increase accelerated from 0.61% during 1999–2016 to 5.77% during 2016–2022. That study also reported higher mortality among males and Black individuals and in the Midwest [13]. The concordance between these results and our pancreatic cancer-specific findings suggests that the post-2016 acceleration may partly reflect a broader increase in cancer-associated PE mortality rather than a phenomenon confined to pancreatic cancer. Changes in PE ascertainment, death certification, cancer survival and the population at risk may also have contributed, but these possibilities cannot be distinguished using aggregate mortality data.

The subgroup joinpoints should similarly be interpreted as statistical changes in slope rather than discrete biological events. Non-Hispanic Black mortality accelerated after 2015, non-Hispanic White mortality after 2018, and regional accelerations were identified between 2014 and 2019. The clustering of several joinpoints in the latter part of the study period is compatible with broader changes in treatment exposure, diagnostic intensity, PE ascertainment, and the population at risk, whereas differences in their timing argue against a single uniform explanation. The particularly steep metropolitan 2018–2020 segment is based on a short terminal interval and should therefore be interpreted cautiously.

The association between pancreatic cancer and thromboembolism is well established. Pancreatic cancer has among the highest VTE incidence rates of any solid tumor [23,24], and prospective studies demonstrate associations between VTE and shorter progression-free and overall survival [4,25,26]. Menapace et al. similarly reported an association between thromboembolic events and mortality in pancreatic adenocarcinoma [27], while population-based data have linked cancer-associated VTE with reduced survival [28]. PE may therefore function both as a direct clinical complication and as a marker of advanced or biologically aggressive malignancy. The present proportional analysis is compatible with increasing PE involvement in the mortality record but cannot establish whether PE directly caused death or instead identified patients with greater tumor burden, treatment-related complications, or other adverse clinical features.

The expanded race/ethnicity analysis clarifies the scope of the disparity findings. Non-Hispanic Black individuals had the highest stable full-period AAMRs, consistent with previously described disparities in pancreatic cancer mortality and cancer-associated VTE [2,29]. Hispanic or Latino mortality increased significantly over the contiguous 2011–2024 interval. Asian or Pacific Islander and American Indian or Alaska Native estimates were also examined; however, frequent suppression and sparse annual counts precluded reliable longitudinal trend modeling. These data limitations restrict comparisons across smaller racial/ethnic populations and mean that the race/ethnicity findings should not be interpreted as exhaustive comparisons or as evidence of inherent biological differences. Potential contributors include stage at diagnosis, comorbidity, treatment access, socioeconomic conditions, structural inequities, and differential ascertainment, none of which are captured in CDC WONDER.

Regional and state-level variation was also considerable. Prior work has associated rural residence and socioeconomic disadvantage with poorer pancreatic cancer survival [30]. In the present analysis, metropolitan areas had a higher AAMR than nonmetropolitan areas by 2020, whereas the broader national analysis of PE mortality across all cancers reported higher rates in rural areas [13]. Differences in pancreatic cancer incidence, referral patterns, imaging intensity, treatment availability, population structure, and death-certification practices may contribute to this apparent divergence. State-level estimates should be interpreted cautiously because some were based on relatively small numbers and some recent estimates were suppressed.

The clinical implication of these findings is therefore one of epidemiologic signal detection rather than a direct treatment recommendation. Existing clinical evidence supports individualized assessment of VTE risk and evidence-based prophylaxis in appropriately selected patients with pancreatic cancer [8,9,18,19,20]. However, the present mortality analysis does not test a risk-assessment strategy, measure anticoagulant use, or demonstrate that additional PE screening or thromboprophylaxis would reduce mortality. Patient-level studies linking tumor stage, systemic therapy, imaging, anticoagulation, bleeding outcomes, and adjudicated PE events are needed to identify the factors underlying these population-level trends.

The ARIMA forecasts should be interpreted as projections rather than predictions of a fixed future trajectory. They extrapolate patterns observed during 1999–2024 and cannot account for future changes in pancreatic cancer incidence, systemic therapy, thromboprophylaxis, PE detection, coding, or clinical management. Each overall and subgroup series was modeled independently; therefore, subgroup forecasts are not expected to aggregate mathematically to the overall projection. Widening prediction intervals further emphasize uncertainty over the longer forecasting horizon.

This study has several strengths, including U.S. population coverage, a 26-year observation period, age-standardized estimates, formal Joinpoint analysis, expanded race/ethnicity evaluation, a proportional analysis distinguishing PE involvement from overall pancreatic cancer mortality, and sensitivity analysis addressing the COVID-19 period. Residual diagnostic testing did not identify significant autocorrelation in the selected ARIMA models. Important limitations remain. Death certificates may contain misclassification or incomplete reporting, and the presence of both diagnoses does not establish their temporal or causal relationship. The data cannot identify tumor stage, systemic therapy, thromboprophylaxis, recurrent thrombosis, incidental versus symptomatic PE, COVID-19 infection, individual comorbidities, socioeconomic status, or access to care. Estimates for Asian or Pacific Islander and American Indian or Alaska Native populations were frequently suppressed or based on sparse counts, preventing reliable longitudinal trend analysis in these groups. Urbanization data were unavailable after 2020, and some state-level estimates were unstable or suppressed. Finally, the ecological design precludes patient-level causal inference.

## 5. Conclusions

Mortality involving pancreatic cancer and PE increased substantially in the United States between 1999 and 2024, with a marked acceleration after 2016. PE was also recorded in a growing proportion of deaths for which pancreatic cancer was the underlying cause, indicating that the joint mortality trend was not explained solely by increasing pancreatic cancer burden. Disparities by sex, race/ethnicity, and geography persisted. Because death-certificate data cannot establish causality, these findings should be interpreted as a population-level epidemiologic signal that warrants patient-level investigation of disease stage, treatment, PE ascertainment, thromboprophylaxis, access to care, and other potentially modifiable contributors.

## Data Availability

The original contributions presented in this study are included in the article/Supplementary Material. Further inquiries can be directed to the corresponding author.

## Supplementary Materials

The following supporting information can be downloaded at: https://www.mdpi.com/article/10.3390/diseases14080303/s1. Table S1. Additional race/ethnicity age-adjusted mortality rates per 100,000; Table S2. Proportion of pancreatic cancer underlying-cause deaths with pulmonary embolism recorded; Table S3. State abbreviation key for Figure 5; Table S4. ARIMA residual autocorrelation diagnostics; Table S5. Summary of added sensitivity and supplementary trend analyses.
