# Supplementary material for: Trends and Disparities in Mortality Involving Pancreatic Cancer and Pulmonary Embolism in the United States, 1999–2024: A CDC WONDER Analysis
Source: Diseases. 2026 Aug 20;14(8):303. doi: 10.3390/diseases14080303 (PMC13511829; doi:10.3390/diseases14080303)
Supplement: Supplementary file 1 [file diseases-14-00303-s001.zip › diseases-4453796-supplementary.pdf]

Supplementary Material

Trends and Disparities in Mortality Involving Pancreatic Cancer and Pulmonary Embolism in the United States, 1999–2024: A CDC WONDER Analysis

Supplementary Methods. Additional race/ethnicity, PE-share, COVID-19 sensitivity, and diagnostic analyses

Race/ethnicity estimates were expanded using the new CDC WONDER exports. Hispanic or Latino AAMRs were summarized from 2011–2024, the first contiguous interval in the supplied exports with reportable annual values suitable for trend analysis. Asian or Pacific Islander and American Indian or Alaska Native estimates were also examined; however, annual estimates were frequently suppressed or based on sparse counts, precluding reliable longitudinal trend analysis. Therefore, APCs were not estimated for these groups, and no suppressed values were imputed. The PE-share analysis required pancreatic cancer to be the underlying cause of death and identified PE among the multiple causes. For the PE-share analysis, the 1999–2020 and 2018–2024 extracts were identical in the overlapping years 2018–2020, permitting a continuous 1999–2024 series. The COVID-19 sensitivity analysis excluded 2020–2021 when re-estimating the post-2016 overall slope. ARIMA residual autocorrelation was assessed with Ljung–Box tests.

Supplementary Table S1. Additional race/ethnicity age-adjusted mortality rates per 100,000

Year	Hispanic or Latino
2011	0.20
2012	0.15
2013	0.19
2014	0.15
2015	0.20
2016	0.21
2017	0.21
2018	0.17
2019	0.28
2020	0.27
2021	0.31
2022	0.30
2023	0.29
2024	0.38

Note: Asian or Pacific Islander and American Indian or Alaska Native estimates were examined, but annual values were too sparse or frequently suppressed to support a stable longitudinal trend series. Therefore, APCs were not estimated for these groups and suppressed values were not imputed.

Supplementary Table S2. Proportion of pancreatic cancer underlying-cause deaths with pulmonary embolism recorded

Year	All pancreatic cancer deaths	With PE recorded	Proportion (%)
1999	29,077	292	1.00
2000	29,322	291	0.99
2001	29,797	320	1.07

2002	30,255	307	1.01
2003	30,766	341	1.11
2004	31,760	319	1.00
2005	32,754	405	1.24
2006	33,446	439	1.31
2007	34,110	459	1.35
2008	35,225	491	1.39
2009	35,617	492	1.38
2010	36,877	524	1.42
2011	37,339	558	1.49
2012	38,789	604	1.56
2013	38,984	590	1.51
2014	40,403	561	1.39
2015	41,601	667	1.60
2016	42,751	676	1.58
2017	44,007	779	1.77
2018	44,903	821	1.83
2019	45,877	916	2.00
2020	46,765	999	2.14
2021	47,897	1,145	2.39
2022	48,316	1,233	2.55
2023	49,441	1,296	2.62
2024	51,313	1,464	2.85

Supplementary Table S3. State abbreviation key for Figure 5

Abbreviation	State	Abbreviation	State
AL	Alabama	MT	Montana
AK	Alaska	NE	Nebraska
AZ	Arizona	NV	Nevada
AR	Arkansas	NH	New Hampshire
CA	California	NJ	New Jersey
CO	Colorado	NM	New Mexico
CT	Connecticut	NY	New York
DE	Delaware	NC	North Carolina
DC	District of Columbia	ND	North Dakota
FL	Florida	OH	Ohio
GA	Georgia	OK	Oklahoma
HI	Hawaii	OR	Oregon
ID	Idaho	PA	Pennsylvania
IL	Illinois	RI	Rhode Island
IN	Indiana	SC	South Carolina
IA	Iowa	SD	South Dakota
KS	Kansas	TN	Tennessee
KY	Kentucky	TX	Texas
LA	Louisiana	UT	Utah
ME	Maine	VT	Vermont
MD	Maryland	VA	Virginia
MA	Massachusetts	WA	Washington
MI	Michigan	WV	West Virginia
MN	Minnesota	WI	Wisconsin
MS	Mississippi	WY	Wyoming
MO	Missouri		

Supplementary Table S4. ARIMA residual autocorrelation diagnostics

Series	Selected model	Ljung–Box lag	P value
Overall	ARIMA(0,2,2)	6	0.260
Female	ARIMA(0,2,2)	6	0.846
Male	ARIMA(0,1,0) with drift	6	0.548
Non-Hispanic Black	ARIMA(1,1,0) with drift	6	0.212
Non-Hispanic White	ARIMA(3,2,0)	6	0.737
Northeast	ARIMA(0,1,0) with drift	6	0.491
Midwest	ARIMA(0,1,0) with drift	6	0.396
South	ARIMA(0,2,2)	6	0.402
West	ARIMA(1,1,0) with drift	6	0.710
Metropolitan	ARIMA(0,1,0) with drift	5	0.993
Nonmetropolitan	ARIMA(0,1,1) with drift	5	0.760

All P values were >0.05, providing no evidence of material residual autocorrelation at the assessed lags.

Supplementary Table S5. Summary of added sensitivity and supplementary trend analyses

Analysis	Estimate	95% CI	P value
Hispanic or Latino, 2011–2024 weighted log-linear APC	6.28% per year	4.51 to 8.08	<0.001
Overall post-2016 slope excluding 2020–2021	8.43% per year	7.33 to 9.53	<0.001
PE-share annual odds change, 1999–2016	2.94% per year	2.56 to 3.33	<0.001
PE-share annual odds change, 2016–2024	7.37% per year	6.69 to 8.06	<0.001
Difference in PE-share slopes at 2016	Post-2016 steeper	—	<0.001
